# Experimental evolution under varying sex ratio and nutrient availability modulates male mating success in *Drosophila melanogaster*

**DOI:** 10.1098/rsbl.2021.0652

**Published:** 2022-06-01

**Authors:** Irem Sepil, Jennifer C. Perry, Alice Dore, Tracey Chapman, Stuart Wigby

**Affiliations:** ^1^ Department of Zoology, University of Oxford, 11a Mansfield Road, Oxford OX1 3SZ, UK; ^2^ School of Biological Sciences, University of East Anglia, Norwich Research Park, Norwich NR4 7TJ, UK; ^3^ Department of Evolution, Ecology, and Behaviour, Institute of Infection, Veterinary and Ecological Sciences, University of Liverpool, Liverpool, UK

**Keywords:** experimental evolution, sexual selection, sex ratio, nutrient availability, pre-copulatory mating traits, post-copulatory mating traits

## Abstract

Biased population sex ratios can alter optimal male mating strategies, and allocation to reproductive traits depends on nutrient availability. However, there is little information on how nutrition interacts with sex ratio to influence the evolution of pre-copulatory and post-copulatory traits separately. To address this omission, we test how male mating success and reproductive investment evolve under varying sex ratios and adult diet in *Drosophila melanogaster,* using experimental evolution. We found that sex ratio and nutrient availability interacted to determine male pre-copulatory performance. Males from female-biased populations were slow to mate when they evolved under protein restriction. By contrast, we found direct and non-interacting effects of sex ratio and nutrient availability on post-copulatory success. Males that evolved under protein restriction were relatively poor at suppressing female remating. Males that evolved under equal sex ratios fathered more offspring and were better at supressing female remating, relative to males from male-biased or female-biased populations. These results support the idea that sex ratios and nutrition interact to determine the evolution of pre-copulatory mating traits, but independently influence the evolution of post-copulatory traits.

## 1. Introduction

The sociosexual environment can profoundly impact the strength and direction of sexual selection. Variation in the sex ratio alters the intensity of intra-sexual competition for mating opportunities and subsequent fertilization. Under male-biased (MB) sex ratios, heightened male–male competition is expected to select for male strategies that increase a male's likelihood of securing a mating [1,2]. However, elevated polyandry—which typically occurs under MB sex ratios—can weaken pre-copulatory sexual selection on males, while strengthening post-copulatory selection [3]. Consequently, males are expected to increase their investment in ejaculate under a MB sex ratio to ensure reproductive success in the presence of sperm competition [4–6]. Under a female-biased (FB) sex ratio, relaxed sexual selection is expected to result in reduced investment in competitive male adaptations for achieving high mating success and paternity.

Experimental evolution is a powerful approach for investigating the evolution of reproductive strategies in response to varying sex ratios [7–10]. In agreement with theory, previous studies found that males from MB populations mated for longer, consistent with higher ejaculate investment [11,12]. Likewise, MB males became ejaculate-depleted faster when mating multiply, suggesting elevated ejaculate investment in earlier matings at the expense of future reproductive performance [8,13]. Yet, in other studies, males from FB populations sired as many or more offspring than MB males, and MB males were slower to start mating compared with males from FB and equal (EQ)-sex populations [1,12,14].

These studies have shed light on evolved responses to the sociosexual environment. However, the evolution of male reproductive strategies is also likely to depend on the nutritional environment. In *Drosophila melanogaster*, males maintained on low-protein diets are poorer at securing matings [15]. Likewise, the impact of nutrient limitation on ejaculate investment is well established. A recent comparative study revealed that investment in seminal fluid protein production is highly sensitive to nutrient availability, while sperm quantity is moderately affected [16]. Hence, the evolution of costly reproductive strategies is likely to be affected by the interaction between nutrient availability and the social environment [17]. However, this interaction has been rarely tested (but see [12,18]).

To examine how male reproductive strategies and investment in pre- and post-copulatory traits evolve under varying sexual selection and nutrient availability, we used experimental evolution in *D. melanogaster.* Sexual selection and nutrient availability were manipulated by varying the adult sex ratio (MB, EQ or FB) and adult nutritional environment (a standard or protein-restricted diet) in replicate populations [12]. Because a MB sex ratio involves intense competition for few mating opportunities, one hypothesis is that MB males should evolve to invest heavily in both securing matings and ejaculate transfer when they do get a mating opportunity, and that they should therefore have the highest reproductive output [19]. Some studies show that pre- and post-copulatory reproductive traits can be positively correlated, while others find no relationship between them [20–23]. An alternative hypothesis is that elevated polyandry in MB populations might weaken selection on the male pre-copulatory traits we tested but strengthen selection on male post-copulatory traits. Previous work has documented that higher polyandry (such as observed in our MB populations) can be associated with low variance in male mating success and hence weak pre-copulatory sexual selection [3,24]. We also predicted that increased investment in reproductive traits would only be possible when nutrients are readily available, so the effects of sex ratio should be limited to populations evolving on a standard-protein diet, especially for traits affected by seminal fluid, such as remating latency and sperm competition, compared to traits mainly affected by sperm [25,26].

## 2. Material and methods

### (a) Experimental evolution

Experimentally evolving populations were derived from an outbred Dahomey (*Dah*) wild-type stock [12,18]. Populations evolved under one of three sex ratio treatments combined with one of two adult dietary regimes in a fully factorial design (18 populations in total). The adult diet treatments were either standard sugar–yeast agar (SYA) medium or a protein-restricted SYA medium containing 20% of the yeast content ([12,18]). A 20% yeast adult diet depresses female lifetime fecundity to approximately 12% of that achieved on a 100% yeast diet [27]. Populations evolved at a MB (70 males : 30 females), EQ (50 : 50) or FB (25 : 75) adult sex ratio. Populations were maintained in non-overlapping generations, established each generation using 100 randomly selected same-aged individuals in the correct sex ratio [12]. Eggs were collected on the 10–11th day of each generation, and larvae were raised at a standardized density on SYA media [18]. Populations were assayed after 35 generations of experimental evolution.

Females and rival males were derived from a stock containing the recessive *sparkling poliert* (*spa*) mutation (frequently used in sperm competition studies [6,22,23]), backcrossed into the *Dah* background for four generations, using more than 50 females and 50 males in each backcrossed generation to limit inbreeding.

### (b) Experimental design

The main experiment was preceded by two generations in which populations were reared under standardized conditions on Lewis medium supplemented with live yeast [28], to reduce variation from parental effects. After two generations, virgin focal (experimentally evolved) males from each population were collected at eclosion and housed in vials in single-sex groups of 12 for 4–6 days. On the day before mating assays, we placed virgin *spa* females (6–8 days old) in individual vials containing Lewis medium supplemented with live yeast. To begin mating assays, we added a focal male to each female vial and observed flies for 320 min. We recorded mating success, latency, and duration. Mated *spa* females were allowed to lay eggs for 2 days within the same vial. We counted emerging offspring 12 days later to measure male reproductive output. After 2 days, we transferred each female into a new vial with two *spa* virgin males (8–10 days old) and gave females a 5-h window in which to remate once. Remating occurrence, latency, and duration were recorded. Remated females were allowed to lay eggs within the same vial for an additional 2 days and we counted and phenotyped the emerging offspring to measure paternity share. Mating and remating trials were conducted over 2 days.

### (c) Statistical analyses

Data were analysed using R v. 3.6.3 [29]. Mating latency, offspring number and remating latency were analysed using a generalized linear mixed effect model (GLMM) with a Poisson error distribution corrected for overdispersion. Mating duration and remating duration were analysed using a linear mixed effects model. Proportion of matings and proportion of rematings were analysed using a GLMM with a binomial error distribution. Paternity share was analysed using a GLMM with a binomial error distribution corrected for overdispersion. The initial model included diet, sex ratio and their interaction and day of the experiment as fixed effects, and replicate population as a random effect. All GLMMs (except binary responses) were initially checked for overdispersion, and when present an observation level random effect was introduced to control for it [30]. All data were analysed using the *lme4* package and model selection was performed by backward stepwise elimination using a maximum-likelihood approach to compare nested models; non-significant (*p* > 0.05) variables were eliminated from the model to arrive at the minimal adequate model. Day and replicate population were retained in the minimal models to control for this variation.

## 3. Results

### (a) Male mating latency and success

**Figure 1.**
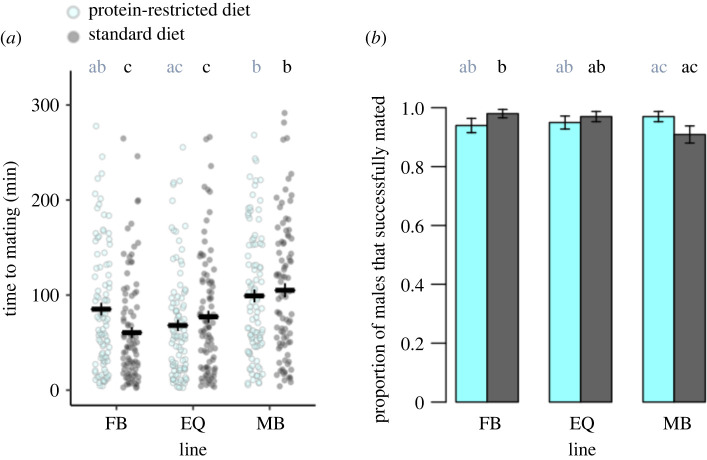
(*a*) Mating latencies and (*b*) mating success of experimentally evolved focal males (mean ± s.e.). Males evolved under FB, EQ or MB sex ratios, and protein-restricted (20% yeast; light blue) or standard (100% yeast; dark grey) diet regimes. Letters indicate significant differences in post hoc tests. For plots of replicate populations and for sample sizes, see electronic supplementary material, figure S1 and table S1.

The interaction between adult sex ratio and diet significantly affected male mating latency ($\chi_{2}^{2}=11.9$; *p* = 0.003). MB males were slower to mate than EQ males regardless of their diet, consistent with previous findings [12] (figure 1*a*). However, diet impacted mating latency in FB males, such that FB males evolved on a standard diet were faster to mate. We also found a marginally significant interaction between sex ratio and diet for male mating probability ($x_{2}^{2}=6$; *p* = 0.048). Among flies that evolved on a standard diet, FB males were more successful than MB males at securing a mating, whereas among flies that evolved on a protein-restricted diet, the mating success of each group was similar (figure 1*b*). In contrast to previous findings [12], we found no effect of either sex ratio or diet on mating duration (sex ratio: $x_{2}^{2}=0.2$; *p* = 0.868; diet: $x_{1}^{2}=0.3$; *p* = 0.551; interaction: $x_{2}^{2}=0.9$; *p* = 0.632) (electronic supplementary material, figure S2).

### (b) Male ability to induce female post-mating responses

**Figure 2.**
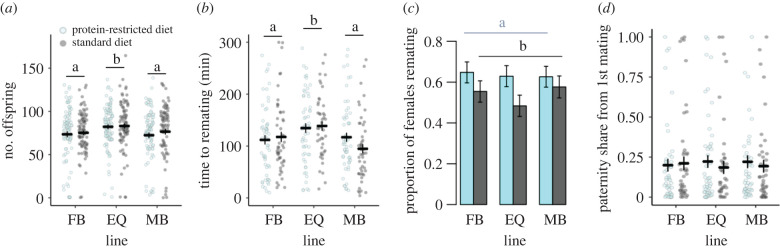
Female post-mating response when wild-type females first mated with experimentally evolved focal males. (*a*) Number of offspring produced in 48 h; (*b*) female remating latencies 48 h following the initial mating; (*c*) remating probabilities 48 h following the initial mating; (*d*) paternity share of the experimentally evolved focal males (mean ± s.e.). Males evolved under FB, EQ or MB sex ratios, and protein-restricted (20% yeast; light blue) or standard (100% yeast; dark grey) diet regimes. Letters indicate significant differences among sex ratio (*a*,*b*) and diet treatments (*c*) in post hoc tests. For plots of replicate populations and for sample sizes, see electronic supplementary material, figures S3–S5 and table S1.

Mates of EQ males produced more offspring, compared to mates of FB and MB males, in the 48 h following a single mating ($\chi_{2}^{2}=6.8$; *p* = 0.033) (figure 2*a*). Neither diet nor its interaction with sex ratio impacted offspring production (diet: $x_{1}^{2}=0.5$; *p* = 0.439; interaction: $x_{2}^{2}=0.9$; *p* = 0.608). Similarly, females that first mated with EQ males took longer to remate compared with females that first mated with FB or MB males ($x_{2}^{2}=7.9$; *p* = 0.018) (figure 2*b*). Remating latency was not influenced by diet or its interaction with sex ratio (diet: $x_{1}^{2}=0.1$; *p* = 0.708; interaction: $x_{2}^{2}=5.3$; *p* = 0.068).

However, we found that a female's probability of remating was impacted by the focal male's diet, such that females that first mated with males that evolved on a protein-restricted diet were more likely to remate ($x_{1}^{2}=5.3$; *p* = 0.02) (figure 2*c*). Remating probability was not affected by sex ratio or the interaction between sex ratio and diet (sex ratio: $x_{2}^{2}=0.6$; *p* = 0.728; interaction: $x_{2}^{2}=0.8$; *p* = 0.653). There was no significant effect of sex ratio, diet or their interaction on either paternity share (sex ratio: $x_{2}^{2}=0.03$; *p* = 0.982; diet: $x_{1}^{2}=0.03$; *p* = 0.571; interaction: $x_{2}^{2}=0.8$; *p* = 0.656) (figure 2*d*) or remating duration (sex ratio: $x_{2}^{2}=0.8$; *p* = 0.655; diet: $x_{1}^{2}=0.2$; *p* = 0.593; interaction: $x_{2}^{2}=2.1$; *p* = 0.335) (electronic supplementary material, figure S6).

## 4. Discussion

In contrast to our first hypothesis, there was no evidence that MB males were better at securing a mating (indicating higher investment in pre-copulatory traits) or increased their post-copulatory investment. However, in line with the alternative hypothesis, we found that MB males were slower to start mating (indicating lower investment in some pre-copulatory traits) and that some effects of sex ratio occurred only when populations evolved on a standard-protein diet.

Our finding that males from FB populations that evolved on a standard-protein diet were quicker to mate and more likely to mate than MB males is consistent with stronger pre-copulatory sexual selection on males in FB populations, possibly through selection on males to secure mating with the most fertile females [31]. Our results also suggest that evolving on a protein-restricted diet led to increased mating latency in FB males, consistent with the hypothesis that male pre-copulatory investment was limited by nutrient availability within FB populations.

Recent work on these populations revealed that MB males respond to exposure to rivals by reducing their courtship behaviour and are hence slower to mate [12], congruent with our findings. This plastic response to rival males before mating is consistent with weakened pre-copulatory sexual selection from increased polyandry and frequent interruption of courtship in MB populations [3,12]. However, under this scenario, we would have expected to find evidence for stronger selection on post-copulatory traits in MB males, but we found no evidence for increases in mating duration (but see [12]), siring success or the inhibition of female re-mating in MB males. It is possible that sex ratio influenced traits not measured in this study, such as male–male aggression or the ability to disrupt rival matings. Future studies that assay these and other traits—such as male ability to achieve mating with resistant females or with virgin females in the presence of other males—would help to expand our understanding of evolutionary responses to sex ratio and nutrition.

In results congruent with ours, Rostant *et al.* [18] found that female resistance to male harm is more likely to evolve in standard-protein regimes, possibly due to the costs involved in expressing resistance. In our experiment, males that evolved on a standard-protein diet were better at suppressing female remating, but were not better sperm competitors. Both suppression of female remating and the ability of a male's sperm to resist displacement by subsequent ejaculates are strongly driven by the transfer of seminal fluid proteins [32,33]. Previous work revealed that female remating propensity and sperm competitiveness are independent and possibly under the regulation of different glandular cell types and a distinct set of proteins [34]. Our results are consistent with the hypothesis that populations that evolved on a protein-restricted diet have altered seminal fluid composition and transfer, with changes limited to a subset of ejaculate functions. To support this interpretation, it will be crucial to investigate the seminal fluid composition of each population in future studies.

It is worth noting that in these experiments we housed flies on molasses-based food media that was distinct from the standard or protein-restricted SYA medium that they evolved on (though likely more similar to the standard than the protein-restricted diet). Dore *et al.* [12] recently used the experimentally evolved populations to test contributions of evolutionary diet and immediate diet (SYA medium with live yeast supplementation, standard SYA without yeast, or protein-restricted SYA) to male mating behaviour and found no influence of immediate diet, suggesting that effects of evolutionary diet are likely to be consistent across variable immediate food types. However post-copulatory traits affected by seminal fluids might respond to the interaction between immediate and evolutionary diet, and future studies should assess flies on both standard and protein-restricted SYA medium to test this.

We used a single competitor and female type (bearing a *spa* eye marker), and it would be interesting to compare against other competitor and female types, and within populations, because the expression of reproductive phenotypes might depend on coevolved interactions between the sexes. For example, a recent study of these populations found that female post-mating aggression is influenced by sex ratio, but only after matings within populations [35].

We found that EQ males were more successful with wild-type females than were MB and FB males in several reproductive traits, including offspring number and remating latency. Several hypotheses might explain this pattern. If wild-type females have an inverted U-shaped preference function for male reproductive traits, such that an intermediate optimum is favoured, then males from MB and FB populations that have evolved away from that optimum might suffer a disadvantage in courting and mating with wild-type females. Males from EQ populations that are more similar to wild-type populations (which have an approximately EQ sex ratio [36]) might gain a relative advantage. An alternative hypothesis, that the observed effects are due to genetic drift or inbreeding, is unlikely because the effective population sizes of these regimes differ only slightly between sex ratio treatments [37].

We found no difference in mating duration among experimentally evolved populations. This is consistent with some previous experimental evolution studies [13,14], but inconsistent with two others, in which MB males mated for significantly longer than FB or EQ males [12,38]. Mating duration has been widely used as an indicator of ejaculate investment in *D. melanogaster*; however, it is becoming increasingly clear that it is not always a good proxy for ejaculate transfer [19,39–41]. Although we found no differences in mating duration, populations differed in traits that suggest differential ejaculate investment, such as offspring number, remating latency and remating success, supporting the idea that there is no association between these measures and mating duration.

Despite the higher productivity and remating suppression of EQ males, we found that paternity share was similar among treatments. This result is consistent with previous work [14] and might be explained by the low heritability of sperm competitiveness and the fact that sperm competition is still present within each sex ratio treatment [42].

In summary, our results show that consistent long-term changes in the socio-sexual and nutritional environment interact to drive the evolution of pre-copulatory traits such as mating latency and success yet independently influence the evolution of post-copulatory traits, such as offspring number and remating latency.

## Data Availability

The complete raw dataset is available on Oxford University Research Archieve (ORA) at the following link https://doi.org/10.5287/bodleian:o1BVXkGQ0. A description of the raw dataset columns is presented in the electronic supplementary material, table S2 [43].
